# Ophthalmia1Read before a meeting of the Wisconsin Society of Veterinary Graduates, March 14, 1900

**Published:** 1900-04

**Authors:** G. Ed. Leech

**Affiliations:** Milwaukee, Wis.


### OPHTHALMIA.[1]

By G. Ed. Leech, M.D.C.,
MILWAUKEE, WIS.

When the subject of periodic ophthalmia came up at the last meeting I little thought that I would be called upon to write a paper upon the subject, and when the Secretary wrote me in regard to it I did not have any great desire to take it up; for it is with some such feeling of dissatisfaction that I do it as must fill the mind of a geographer who is about to enter upon the description of a country into the heart of which no traveller has ever yet been able to penetrate; may we be able to set about the description of a disease whose nature and cure even yet remain to be developed.

It would be asserting too much to say that we do not understand more about it than the former practitioners of "horse medicine" did. Science has shed its light upon and much improved our knowledge of diseases of the eye as well as those of other organs, but all art and practice have failed to furnish us with anything in the shape of a remedy by which we are able to arrest this one in its destructive course, or prevent its almost sure return and fatal termination, and, therefore, in point of naked fact, what we have professed to learn concerning periodic ophthalmia has turned out to be of very little practical use to us.

Still it is our duty to record what we do know, to lay down such rules for the guidance of future investigators as our experience has put us in possession of, and with such views as these, rather than with great prospects of proving of much benefit to our suffering patients, must we enter upon the subject of this paper.

This is, indeed, as has been said of some other diseases, "the bane of horseflesh." What can be more annoying to the feelings of the owner of a good horse than to be told that what he took to be simply a cold in the eye, or a weak eye, is likely, nay, almost sure, to prove in the end a case of blindness, and as it is not within the skill of his medical adviser to prevent this fatal termination he had better avail himself of the first opportunity to dispose of him.

[1] Read before a meeting of the Wisconsin Society of Veterinary Graduates, March 14, 1900

Such advice as this is enough to make him exclaim: "Throw physics to the dogs, I want none of it."

*Name.* Among the various appellations that have been applied to it we are still using the one given by the French veterinarian who preferred it on account of its relapses, as though it were a fresh disease, after having been absent for a more or less considerable time. Professor Coleman called it specific ophthalmia, the same being applicable to glanders, grease, azoturia, etc. The earlier writers called it moon-blindness or lunatic blindness, claiming that as the moon changed the horse gradually recovered its sight. Some also called the affection "moon-eyes." Mr. Fearon called it gouty ophthalmia; Mr. Spooner, hereditary ophthalmia, and Mr. Croxford asks why it may not be called constitutional ophthalmia, and goes on to say that after all the knowledge we have on the subject this seems to him the one most appropriate to use.

*Symptoms.* The symptoms, together with the history attached to them, are sufficient to give the practitioner a correct idea of the nature and progress of the disease. One of the most common accounts one gets is that the horse was all right the night before, and that something must have got into the eye during the night; and, indeed, the half-closed aspect only verifies the statement. The upper lid droops on the corner to shut out the light; tears are produced in such quantity that they cannot be carried off by the puncta lachrymalis, and as a consequent result flow over the lower lid and stream down the face; both eyelids, together with the venous vessels in the immediate vicinity of the eyes, are tumid and fuller than ordinarily. What little is visible of the globe of the eye appears dull and sunken, the organ is very intolerant to light, and upon reflecting the lids, causing the ejection of the membrana nictitans, we are given a view of the membrana conjunctiva reddened and inflamed, commonly of the subacute character, and more or less tumid from infiltration. The circumference of the cornea sometimes exhibits a broad nebulous circle, being an extension of that which in the human is called *arcus senilis*.

In the human eye where there is an *arcus senilis* of the cornea a similar opaque ring exists around the margin of the crystalline body. Is this the case in the horse under disease? At the beginning the anterior chamber of the eye commonly preserves its lucidity, so that we distinctly view the iris and pupil through it, the latter much contracted, the former unchanged in color; but in the course of two or three days afterward, and sometimes on the very day of the attack, the chamber becomes obscured by a dingy white or amber colored deposit seen floating within it, through which the pupil is hardly discernible, contracted, as it is to the breadth of a broad line and looking more like the black eye of a garden bean than the ovoid aperture it was before. Supervening upon this we in some cases have obscuration of the cornea taking place, arising from an extension of the conjunctival inflammation over it, and this in very severe cases is so intense that the vessels carrying red blood are perceptible upon its surface, shooting from all sides of the circumference into a sort of circulus vasculosus, from which others proceed after the manner of radii toward a common centre. The obscuration of the cornea, though it may still leave the lymph effused into the chamber of the eye visible, precludes us from distinguishing the pupil and the iris, and it is not until the inflammation has abated that we again regain a view of those parts, and this constitutes the first or inflammatory stage of ophthalmia, which generally lasts from three to ten days or longer, according to the intensity of the inflammation, differing only in different stages. In the third or fourth stage we see through the anterior chamber the iris, murky and darkened, altered in color and lustreless, with the pupil contracted as much as ever, but not evincing that sensitiveness to light which it did in the inflammatory stage.

Within the chamber and gravitating toward the bottom of it are to be discerned flakes or flocculi of whitish or yellowish lymph, effusions as we suppose from the vessels which secrete the aqueous humor. There is no longer the overflow of tears on the face or the conjunctival inflammation, showing clearly that the inflammatory stage is passed, leaving only its consequences behind, and the eye appears to recover from day to day until there is a relapse again. Relapses are looked for as a matter of course, and yet there are cases where there has been but one attack, and that one not of destructive nature.

The changes that the structure of the diseased eye undergoes are with few exceptions the result of the excessive inflammation, and in general require some considerable time for its completion. The first attack, mild in its nature and not of long duration, may leave the eye altered only in such respects as in the course of a long intermission may be rectified. Commonly after the second attack, and occasionally after the first, there will still remain more or less haziness of the cornea, through which we perceive the iris lustreless and murky in its aspect, the pupil contracted and without any of its natural bright blue to be seen. The corpora nigra also appears more pendulous than usual, wanting their jetty blackness, and on occasions exhibiting light specks of opacity, and every subsequent attack will add to these structural changes.

*Morbid Anatomy.* D'Arbovals, the eminent French veterinarian, says that observation shows an absence of any distinct chambers containing aqueous humor, nothing, in fact, but a single cavity remaining; sometimes the iris appears lacerated, detached from the lens, reduced to a very small volume, its capsule only remaining, perhaps thickened and opaque; at other times the lens is of the natural size, its capsule being opaque with some white spots in its substance and concretions upon its inner surface. The posterior portion of the crystalline lens is thickened and indurated, almost as if it had been boiled, and it reflects a bottle-green color. The fibres of the iris surrounding the lens on some occasions become osseous, and in the place of the vitreous humor resulting from its decomposition we find a viscus orange-colored fluid heavier than water. Instead of the retina there is a fibrous membrane behind the crystalline, the optic nerve is flabby and softened.

Geldings are more liable to suffer from this disease than mares, and by some writers it is supposed that there is some connection between dentition and ophthalmia. The eye most subject to this disease is the very small dark-looking one, which does not disclose any appearance of the white, wall-eyes or watch-eyes seeming to be exempt.

*Causes.* The causes of periodic ophthalmia demand the greatest attention from us, both on account of the light which they shed upon the nature of the disease and the suggestions they furnish for its prevention. Hereditary influence is one of the causes which some of the profession claim it arises from, and no doubt it can be well sustained, for the records show that in those countries where there is no restriction as to what can be used for breeding they have a larger percentage of the disease; but there is some doubt in my mind as to whether in this case it is the disease that is transmitted or only the predisposition. If this is answered "Yes," how, then, is it that the disease so much confines itself to horses of certain ages and situations? If "No" is the answer, then ask yourself why the production of the disease under certain conditions and states of excitement can be most satisfactorily accounted for? I am still firmly of the opinion that it is predisposition only and not excitant.

We now come to the question whether this disease is constitutional or local, or, in other words, is it due to some microörganism in the blood or is the predisposition inherent in the formation or excitability of the eye? These are most interesting questions of which various facts and observations current among us may be brought forward by investigation. But it matters not whether this is called hereditary or due to an original predisposition, it amounts to one and the same thing, and I will venture to say that this is by far the most frequent cause of the disease; but while I make this assertion I stand ready to admit that it also arises from a variety of other causes, quite adventitious and unconnected with this source. Among these may be mentioned indigestion, miasma, neuritis, dentition, debility from overwork, depletion from blood-letting, high-feeding, too much confinement, and sudden exposure to light. It is also a very noticeable fact that those horses not accustomed to domestication are, generally speaking, free from it, and it is noticed to be more prevalent in poorly-ventilated stables, the same as other constitutional diseases.

To what, then, is ophthalmia to be attributed? The advocate of hereditary influence answers, to the circumstance of the parent having had it, while others will say "No," but to nervous influence, to heat, to plethora, to a contaminated atmosphere. For my own part, however, much heredity or other causes may predispose the animal to take the disease. I cannot help thinking that many horses which now contract ophthalmia in stables would escape in situations in the open air, and that in stables we find the number of cases proportionately less according as the animal at the trying time of life (between four and six years) is moderately fed and worked and kept in an uncontaminated atmosphere. I believe that anything that will excite commotion in the system at this period is liable to affect the eyes, though the eye is not so liable to be affected as the membrane lining the air passages. Most young horses at this time of life, on being stabled, are sure to be afflicted with some catarrhal or bronchitic trouble, as strangles, influenza, swollen limbs, diarrhœa, etc. I cannot, therefore, after viewing these cases from all sides, hesitate in pronouncing this disease constitutional and not local—not a single conjunctival inflammation, although the conjunctiva is a participant in it, but essentially and primarily a disease of the internal structure of the eye. Just how these structures become affected I cannot decidedly affirm, but only venture upon an opinion that the blood is the medium of contamination, which is only one opinion among many others just as deserving.

The treatment for this disease is very unsatisfactory, and I will only say that the operation of tapping the aqueous chamber is recorded as early as 1841, and was the result of an accident—recorded by Dr. Price, of Cork.

(To be continued.)

